# Resistance phenotype and virulence potential of Leclercia adecarboxylata strains isolated from different sources

**DOI:** 10.1099/mic.0.001457

**Published:** 2024-04-25

**Authors:** Viviana Yescas-Zazueta, Rosa del Carmen Rocha-Gracia, Cesar R. González-Bonilla, J. Fernando Ayala-Zavala, Yessica Enciso-Martínez, Eder A. Carreón-León, Brigitte A. González Corona, Dora Valencia, Manuel G. Ballesteros-Monrreal, Edwin Barrios-Villa

**Affiliations:** 1 Departamento de Ciencias Químico Biológicas y Agropecuarias, Universidad de Sonora, campus Caborca, Av. Universidad e Irigoyen S/N, 83621 H. Caborca, Sonora, Mexico; 2 Posgrado en Microbiología, Centro de Investigaciones en Ciencias Microbiológicas, Instituto de Ciencias Benemérita Universidad Autónoma de Puebla, Av. San Claudio S/N, 72570 Puebla, Mexico; 3 Coordinación de Tecnología de Alimentos de Origen Vegetal, Centro de Investigación en Alimentación y Desarrollo, A.C., 83304 Hermosillo, Mexico; 4 Laboratorio de Análisis Clínicos de la Universidad Autónoma de Chihuahua, Av. Universidad S/N, Circuito Universitario Campus 1, 31310 Chihuahua, Mexico

**Keywords:** emerging pathogen, *L. adecarboxylata*, plasmids, resistance, virulence

## Abstract

**Introduction.***Leclercia adecarboxylata* is a member of Enterobacterales, often considered an opportunistic pathogen. Recent reports have highlighted *L. adecarboxylata* as an emerging pathogen harbouring virulence and resistance determinants.

**Gap statement.** Little information exists on virulence and resistance determinants in *L. adecarboxylata* strains isolated from environmental, food, and clinical samples.

**Aim.** To determine the presence of resistance and virulence determinants and plasmid features in *L. adecarboxylata* strains isolated from environmental, food, and clinical samples, as well as their phylogenetic relationship.

**Results.** All strains tested showed resistance to β-lactams and quinolones but were sensitive to aminoglycosides and nitrofurans. However, even though fosfomycin resistance is considered a characteristic trait of *L. adecarboxylata*, the resistance phenotype was only observed in 50 % of the strains; *bla*_TEM_ was the most prevalent BLEE gene (70 %), while the quinolone *qnrB* gene was observed in 60 % of the strains. Virulence genes were differentially observed in the strains, with adhesion-related genes being the most abundant, followed by toxin genes. Finally, all strains carried one to seven plasmid bands ranging from 7 to 125 kbps and harboured several plasmid addiction systems, such as ParDE, VagCD, and CcdAB in 80 % of the strains.

**Conclusions.***L. adecarboxylata* is an important emerging pathogen that may harbour resistance and virulence genes. Additionally, it has mobilizable genetic elements that may contribute to the dissemination of genetic determinants to other bacterial genera.

**Impact Statement**

This study highlights the presence of resistance and virulence determinants in *L. adecarboxylata* strains, their pathogenic potential, and their role as a reservoir of those genetic determinants, as well as the impact of this microorganism as a potential donor of virulence and resistance genetic determinants through horizontal transfer mediated by conjugative plasmids. Additionally, we report the presence of plasmid addiction systems previously reported in virulence-associated plasmids.

This article also highlights the importance of identifying the species *L. adecarboxylata*. The findings presented here reinforce the rationale for considering *L. adecarboxylata* as an emerging pathogen based on its presence in clinical, food and environmental samples.

## Data Summary

The authors confirm all supporting data, code and protocols have been provided within the article or through supplementary data files.

## Introduction

*Leclercia adecarboxylata* is a rod-shaped bacterium found in the environment, food, sewage, animals, and clinical specimens without causing harm [1,2]. It is usually recognized as a harmless bacterium, and when it has been reported in association with a clinical picture, it is often secondary to a primary pathogen [3]. Most cases in the literature classify it as an opportunistic pathogen. It has been reported to cause bloodstream infections, catheter infections, endocarditis, peritonitis, folliculitis, urinary tract infections, and wound infections [3,9]. Recent reports have recognized * L. adecarboxylata* as an important emerging pathogen since it has been a cause of primary infections in immunocompetent patients [10,11]. Infections caused by *L. adecarboxylata* usually respond to first-choice antimicrobial treatments due to its low antimicrobial resistance. However, it presents intrinsic resistance to fosfomycin mediated by the presence of the *fosA*^LA^ gene [12].

Despite this, several papers worldwide have reported the presence of *L. adecarboxylata* strains resistant to multiple antibiotics, making this species an important reservoir of resistance genes that can be transferred intra- and interspecies. In that sense, with a Whole Genome Sequencing approach, our working group recently reported the co-occurrence of resistance genes and virulence genes present in important human pathogens within *L. adecarboxylata* strains, suggesting the need to consider this species as an emerging pathogen [13]. The clinical relevance of *L. adecarboxylata* has been demonstrated since the isolation of this microorganism as causative agent in monomicrobial infections such as wound, conjunctive, urinary tract infections (UTI), and bloodstream infections in immunocompetent patients [2,3, 6,11, 14, 15]. Spiegelhauer *et al*., discussed the need for co-infection of *L. adecarboxylata* due to the poor adherence and invasion capacity to HeP-2 cells, however, they considered important the cytotoxic effect of the bacterium on that cell line [16]. Molecular mechanisms involved in Horizontal Gene Transfer (HGT), including plasmids, transposons, and integrons, play a major role in resistance and virulence genetic determinants [17]. Plasmids confer phenotypic traits involving invasion, genome evolution, host range, transition, and persistence [18]. Plasmids often confer benefits to the receptor strains encoding for metabolites, virulence, or resistance genes, but they present an important metabolic/fitness cost [19]. Molecular mechanisms of host persistence called plasmid addiction systems (PAS) associated with conjugative plasmids have been described and represent an important feature for resistance and virulence propagation. Toxin-antitoxin systems, PemK-PemI (plasmid emergency maintenance), CcdA-CcdB (couple cell division locus), RelB-RelE (relaxed control of stable RNA synthesis), ParD-ParE (plasmid stability and stress response module), VagC-VagD (virulence-associated protein); and plasmid antisense RNA-regulated systems, Hok-Sok (host killing), PndA-PndC (promotion of nucleic acid degradation), and SrnB-SrnC (stable RNA) are the most studied PAS [18,20, 21].

This study aimed to determine the presence of resistance and virulence determinants and plasmid features in *L. adecarboxylata* strains isolated from environmental, food, and clinical samples and to perform a comparative analysis to infer their pathogenic potential.

## Methods

### Strains

A subset of ten *Lelcercia adecarboxylata* strains was recovered from the strain collection of the Departamento de Ciencias Químico Biológicas y Agropecuarias from the Universidad de Sonora. All the strains were biochemically identified using a Vitek 2 System (BioMérieux, France), and their identity was confirmed as previously reported (Fig. S1, available in the online version of this article) [22]. LaC1 and LaC34 were obtained from a bloodstream infection and a parenteral nutrition bottle, respectively; these were associated with a hospital outbreak previously characterized and used as a control [13]. The strains AL5, AL19, AL74, and AL100 were recovered from vegetables (consumed raw) at Chihuahua, Mexico. The strain SM14 was obtained from a fruit-management table surface and the strain T17 was isolated from farmland at Hermosillo, Sonora, Mexico; and the strains 27ATM and 33MEM, were recovered from a urine culture associated with a polymicrobial infection in patients from Caborca, Sonora, Mexico (Table 1). All the strains were biochemically identified using a Vitek2 Compact System (BioMérieux, France); identity was confirmed with conventional PCR with specific oligonucleotides [22,30] (Table S1).

**Table 1.** Characteristics of the *L. adecarboxylata* strains

Strain	Source	Virotype												Resistance genotype			Plasmid Addiction System (PAS)
		Adherence						Toxins				IU	Ie				
		*fimH*	*afa-draBC*	*sfaD/focC*	*papC*	*papG-*II	*fliCD*	*cnf-1*	*hlyA*	*vatA*	*ibeA*	*iroN*	*agn43*	ESBL	Non-ESBL		
LaC1	BSI	−	+	−	−	−	−	−	−	−	+	−	−	*bla*_TEM,_ *bla*_SHV_	*qnrB*	ParD-E, Hok-Sok, VagC-D, CcdA-B	
LaC34	TPN bottle	+	+	+	+	+	+	+	−	+	+	−	+	*bla*_TEM_	*qnrB*	PndA-C, ParD-E, PemK-A,Hok-Sok, CcdA-B	
AL5	Raw vegetables	−	+	−	−	+	+	+	+	+	−	−	+	−	−	VagC-D	
AL19	Raw vegetables	−	+	+	+	+	−	−	−	+	+	−	+	−	*qnrB, qepA, oqxA*	ParD-E, PemK-A, Hok-Sok, VagC-D, CcdA-B	
AL74	Raw vegetables	+	−	+	+	+	+	−	+	+	+	−	+	*bla*_TEM_	*qnrB*	ParD-E, VagC-D	
AL100	Raw vegetables	−	+	−	−	−	−	−	−	−	+	−	+	*bla*_TEM_	*qnrB, oqxA*	ParD-E, Hok-Sok, VagC-D, CcdA-B	
27ATM	Urine culture	+	−	−	−	+	−	−	−	−	+	+	+	*bla*_TEM_	−	ParD-E, Hok-Sok, VagC-D, CcdA-B	
33MEM	Urine culture	+	−	+	+	+	+	−	+	−	+	−	+	*bla*_TEM_	−	ParD-E, VagC-D, CcdA-B	
SM14	Inert surface	+	−	+	−	+	−	−	−	−	+	−	−	*bla*_TEM_	*qnrB, aac(6´)-Ib-cr*	VagC-D, CcdA-B	
T17	Cropland	−	−	+	+	+	+	−	+	+	+	−	+	−	*qnrB, aac(6´)-Ib-cr*	ParD-E, Hok-Sok, VagC-D, CcdA-B	

BSI bloodstream infection CcdA–CcdB coupled cell division locus Ie immune evasion IU iron uptake ParD–ParE Hok–Sok host-killing PemK–PemI plasmid emergency maintenance PndA–PndC promotion of nucleic acid degradation RelB–RelE VagC–VagD virulence associated protein SrnB–SrnC stable RNA TPN total parenteral nutrition; a: isolated from a polymicrobial urine culture

### Genomic DNA extraction

Genomic DNA was obtained using the boiling method [31]. Briefly, a 24 h culture on Luria Bertani (LB) agar plates was harvested in 500 µl of nuclease-free water, boiled for 15 min, and centrifuged at 13000 ***g***. The supernatant was recovered into a new tube. DNA concentration was adjusted to 200 ng ml^−1^ with nuclease-free water.

### Plasmid DNA extraction

Plasmid DNA was obtained following the manufacturer's directions using an 18 h culture in Luria Bertani (LB) broth and the Wizard Plus SV Minipreps DNA Purification System (Promega Corporation, Madison WI, USA).

### Polymerase chain reaction for molecular identification, resistance and virulence genes, and plasmid addiction systems

Each reaction contained 0.4 µM of each primer, GoTaq MasterMix (Promega Corporation, Madison WI, USA) (containing reaction Buffer, 400 µM of each dNTP, MgCl2 3 mM and Taq DNA polymerase 1 x) to a final volume of 12 µl in a MiniAmp Plus thermal cycler (Applied BioSystems, USA). The amplicons were resolved on a 1 % agarose gel electrophoresis and stained with Ethidium Bromide to visualize using a UVP Transilluminator (AnalytikJena, USA). The primers are described in Table S1.

### Determination of minimum inhibitory concentration (MIC)

The strains were tested for MIC according to CLSI specifications [32]. Dilutions of ampicillin (AMP), ampicillin/sulbactam (SAM), cephalotin (DCI), cefuroxime (CXM), ceftriaxone (CRO), cefotaxime (CTX), ceftazidime (CAZ), cefepime (FEP), ertapenem (ETP), meropenem (MEM), amikacin (AMK), gentamicin (G), ciprofloxacin (CIP), norfloxacin (NOR), fosfomycin (FOS), nitrofurantoin (NIT), trimethoprim/sulfamethoxazole (TMP-SMX), and cefoxitin (FOX) in concentrations according with CLSI were used in 100 µl of Müeller Hinton broth distributed in a 96-well plate. A 1 : 20 dilution of a 0.5 McFarland standard of an adjusted preculture of each strain was added to the well. After 18 h of incubation at 37 °C, the plate was read for growth inhibition using a BioTek microplate reader at 492 nm. Each strain was triple-tested and confirmed by Minimum Bactericidal Concentration plating on Müeller Hinton agar plates supplemented with the antibiotic concentration tested.

### ERIC-PCR phylogeny

To establish a phylogenetic relationship, an ERIC-PCR was performed using primers previously reported (Table S1) [33]. The reaction tube contained 0.4 µM of each primer, GoTaq MasterMix (Promega Corporation, Madison WI, USA) (containing reaction Buffer, 400 µM of each dNTP, MgCl2 3 mM and Taq DNA polymerase 1 x) to a final volume of 12 µl in a MiniAmp Plus thermal cycler (Applied BioSystems, USA). The protocol includes a denaturation cycle at 95 °C followed by 35 cycles of denaturation at 95 °C for 60 s, 50 °C for 60 s, and 72 °C for 8 min, and a final cycle of extension for 16 min. The amplicons were resolved on a 1% agarose gel electrophoresis and Ethidium Bromide to visualize using a UVP Transilluminator (AnalytikJena, USA).

The images obtained were processed with GelJ 2.0 software [34] to generate a Newick file. The iTOL 6.8 software [35] processed the Newick file to obtain the dendrogram.

### Biofilm formation assays

Biofilm forming ability was tested on 96-well plates following established protocols with some modifications [31,36]. Bacteria were incubated in LB broth for 24 h at 37 °C. A 1 : 14 dilution was added to each well and was incubated for 24 h at 37 °C. Optical density was determined at 492 nm using a BioTek microplate reader and the software SkanIt version 4.1 (Multiskan GO). After eliminating the supernatant, each well was washed with sterile phosphate buffer. Then 20 µl of 1 % solution of crystal violet was added and incubated for 15 min at room temperature, and the plate was vigorously washed with distilled water. Subsequently, 230 µl of 96 % ethanol was added and incubated for 2 min at room temperature. Optical density (O.D.) was determined at 600 nm. The average of three independent O.D. lectures for each sample was extrapolated to a bovine serum albumin calibration curve.

### Statistical analysis

The significant differences between measures were determined with a two-way ANOVA analysis performed with Bonferroni test, and with a 95 % confident interval with a *p* value<0.01 using GraphPad (v9) from Prism software.

## Results

### *L. adecarboxylata* resistance characteristics include quinolone resistance and ESBL production

**Fig. 1.**
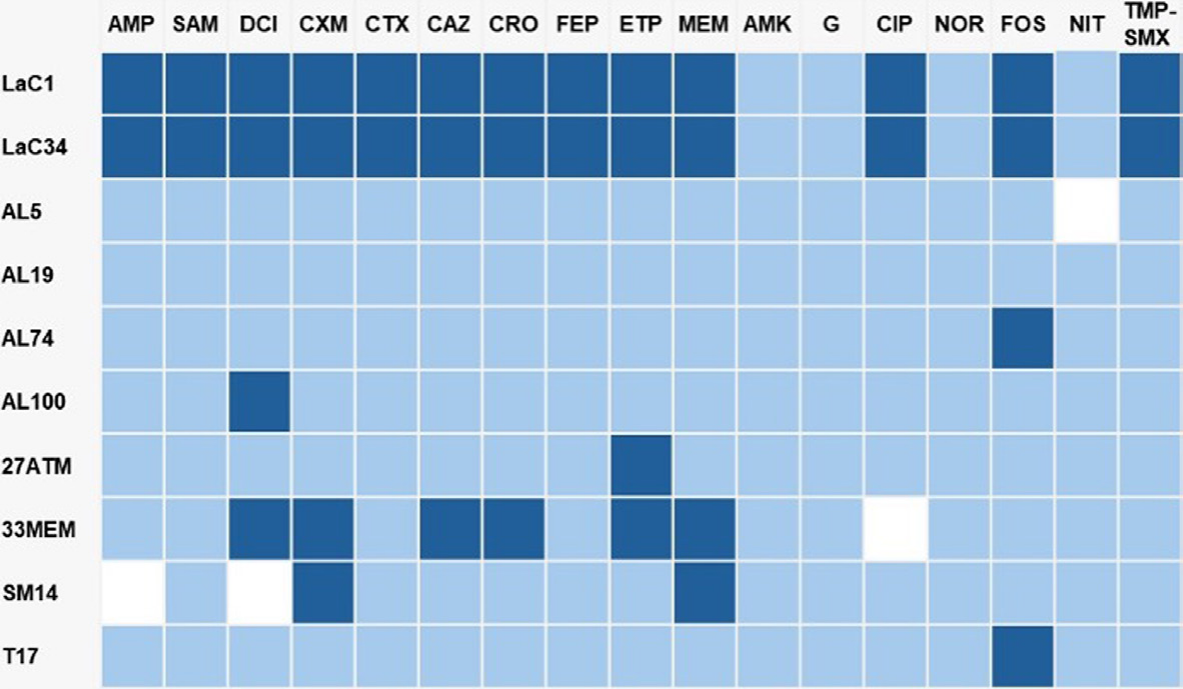
Minimum Inhibitory Concentration-based heatmap of the resistance phenotype presented by the *L. adecarboxylata* strains analysed. Dark blue squares correspond to resistance breakpoints, light blue squares correspond to sensitivity breakpoints, white squares correspond to intermediate sensitivity breakpoint. AMP, ampicillin; SAM, ampicillin/sulbactam; DCI, cephalothin; CXM, cefuroxime; CTX, cefotaxime; CAZ, ceftazidime; CRO, ceftriaxone; FEP, cefepime; ETP, ertapenem; MEM, meropenem; AMK, amikacin; G, gentamicin; CIP, ciprofloxacin; NOR, norfloxacin; FOS, fosfomycin; NIT, nitrofurantoin; TMP-SMX, trimethoprim/sulfamethoxazole. MIC quantifications are provided in Table S2.

The antimicrobial resistance profiles of the *L. adecarboxylata* strains analysed were summarized in Table S2. The strain AL19 was sensitive to all the studied antibiotics; the remaining were resistant to at least two antibiotics. Forty percent (4/10) showed resistance to cephalothin, cefuroxime, and ertapenem; 30 % (3/10) showed resistance to amoxicillin, ampicillin, ceftazidime, ceftriaxone and meropenem; and resistance to ampicillin/sulbactam, cefotaxime, ciprofloxacin and trimethoprim/sulfamethoxazole was shown by 20 % (2/10) of the strains. According to Magiorakos’ classification scheme [37], 30 % (3/10) of the strains were MDR, including those associated with a hospital outbreak (LaC1 and LaC34) and one isolated from a urine sample (33MEM). The strains LaC1 (isolated from blood stream) and LaC34 (isolated from a Total Parenteral Nutrition Bottle) obtained from a hospital outbreak were previously characterized as Multidrug Resistant (MDR) using a WGS approach; phenotypically, both showed resistance to five antibiotic categories. The strains isolated from raw vegetables showed the lowest resistance, strain AL5 presented resistance to amoxicillin and ampicillin, while strain AL74 was resistant to fosfomycin, and AL100 showed resistance to cephalothin. The strains T17 and 27ATM presented a similar behaviour, which showed resistance to fosfomycin and ertapenem respectively. Interestingly, strain 33MEM presented resistance to cephalothin, cefuroxime, ceftazidime, ceftriaxone, ertapenem, and fosfomycin while the strain SM14 showed resistance to cefuroxime and meropenem but presented intermediate sensitivity to ampicillin and cephalothin also (Fig. 1).

### The plasmid load in *L. adecarboxylata* strains is shared among origins and maintained by plasmid addiction systems

The plasmids harboured in the *L. adecarboxylata* strains analysed ranged from 7 to 125 kbs, and heterogeneous distribution of plasmid bands was observed among strains from different origins (Fig. S2). Strains Lac1 and Lac34 have been previously characterized, and clonally related [13] and presented three shared plasmids. In the same sense, strains AL74 and SM14 presented four plasmid bands; this finding is interesting since the strains are from different sources and geographical regions. Another interesting finding was that the strain AL19 presented the highest number of plasmid bands (seven plasmid bands) and showed four with a similar loading pattern with AL74 and SM14 (Fig. S2). The Plasmid Addiction System (PAS) PndA-PndC was found only in the strain LaC34 isolated from a total parenteral nutrition bottle; this strain also harbours ParD-ParE, PemK-PemA, Hok-Sok, and CcdA-CcdB. A similar PAS load was observed for the strain AL19, which presented ParD-ParE, PemK-PemA, Hok-Sok, CcdA-CcdB, and VagC-VagD. Similarly, Lac1, AL100, 27ATM, and T17 strains presented ParD-ParE, Hok-Sok, VagC-VagD, and CcdA-CcdB. On the other hand, the strains 33MEM, SM14, AL74, and AL5 presented three, two, two, and one PAS, respectively (Table 1). 

### Virulence determinants in *L. adecarboxylata* strains are shared with *E. coli*.

Specific PCRs were performed to evaluate the pathogenic potential of the analysed strains for adherence, toxins, iron uptake, and immune evasion. The differential distribution of virulence factors (VF) is summarized in Table 1. The more prevalent VF-associated gene was *ibeA* (90 %), followed by *papG*-II and *agn43* (80 %); the less prevalent VF gene was *iroN,* observed only in the strain 27ATM. The strain showing the highest number of VFs was LaC34 consistent with the previously reported WGS data [13]. Interestingly, the strains recovered from raw vegetables presented a variety of VFs. AL74 harbours 75 % of the VF investigated, including several factors involved in adherence but in toxicity also; in the same way, AL5 and AL19 presented a high load of VF for adherence, cytotoxicity, and immune evasion. Another interesting finding was observed when comparing VFs from urine-recovered strains against those in environmental strains. The strains 33MEM and SM14 carry several genes linked to surface adhesion although 33MEM is more toxigenic.

### The ability to develop biofilm is a characteristic trait in *L. adecarboxylata*.

**Fig. 2.**
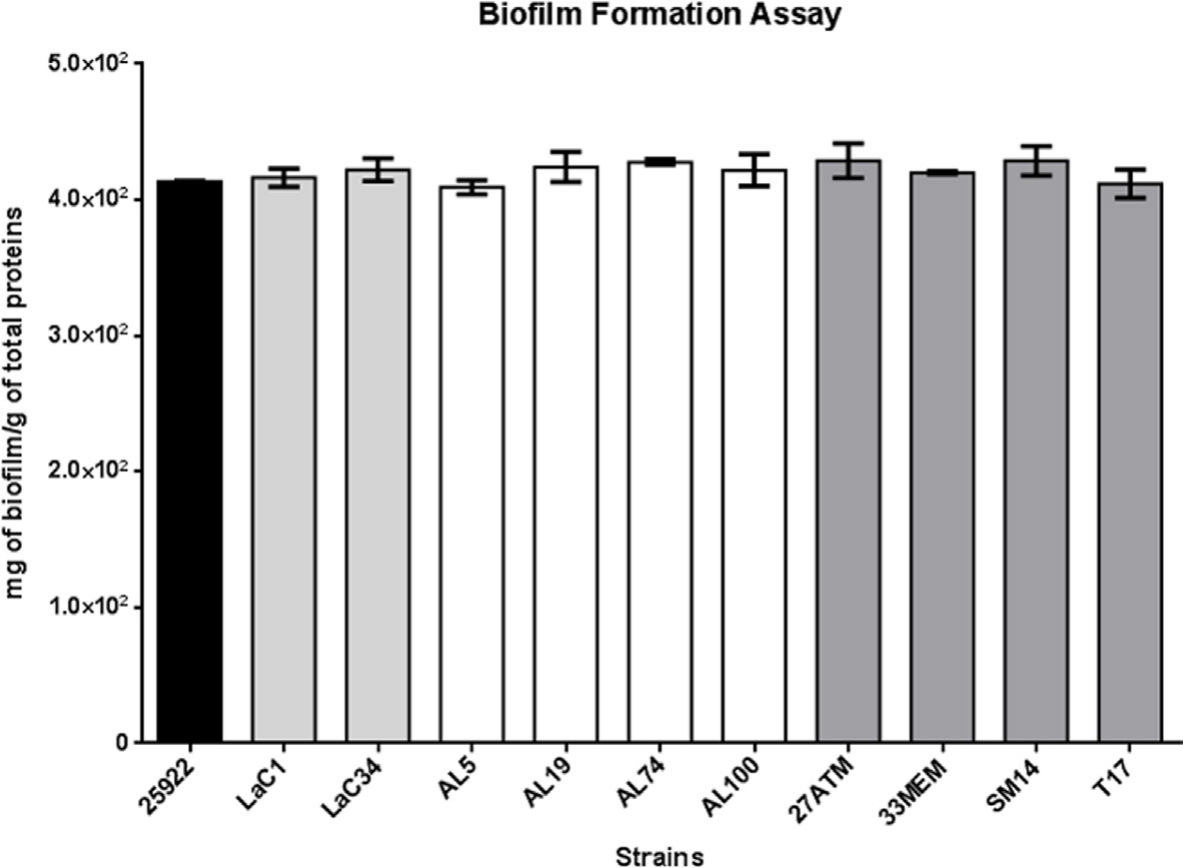
Biofilm formation assays. The average of three assays was interpolated to an albumin calibration curve. The *E. coli* ATCC25922 (black bar) was used as a positive control, the different grey tones represent the source (hospital outbreak, food, urine, and environmental). The bars indicate the standard deviation, and no significative differences were observed in a two-way ANOVA analysis.

Biofilms are produced by lots of different bacterial species, including non-pathogenic ones. Here, we tested this trait for each strain. The biofilm production was consistent (oscillating from 400 to 430 mg of biofilm per gram of total protein) in every strain and is comparable to the production of *E. coli* ATCC25922 (positive control) and has been considered as strong biofilm producer (Fig. 2).

### The *L. adecarboxylata* phylogeny suggests a close relationship among food and nosocomial strains

**Fig. 3.**
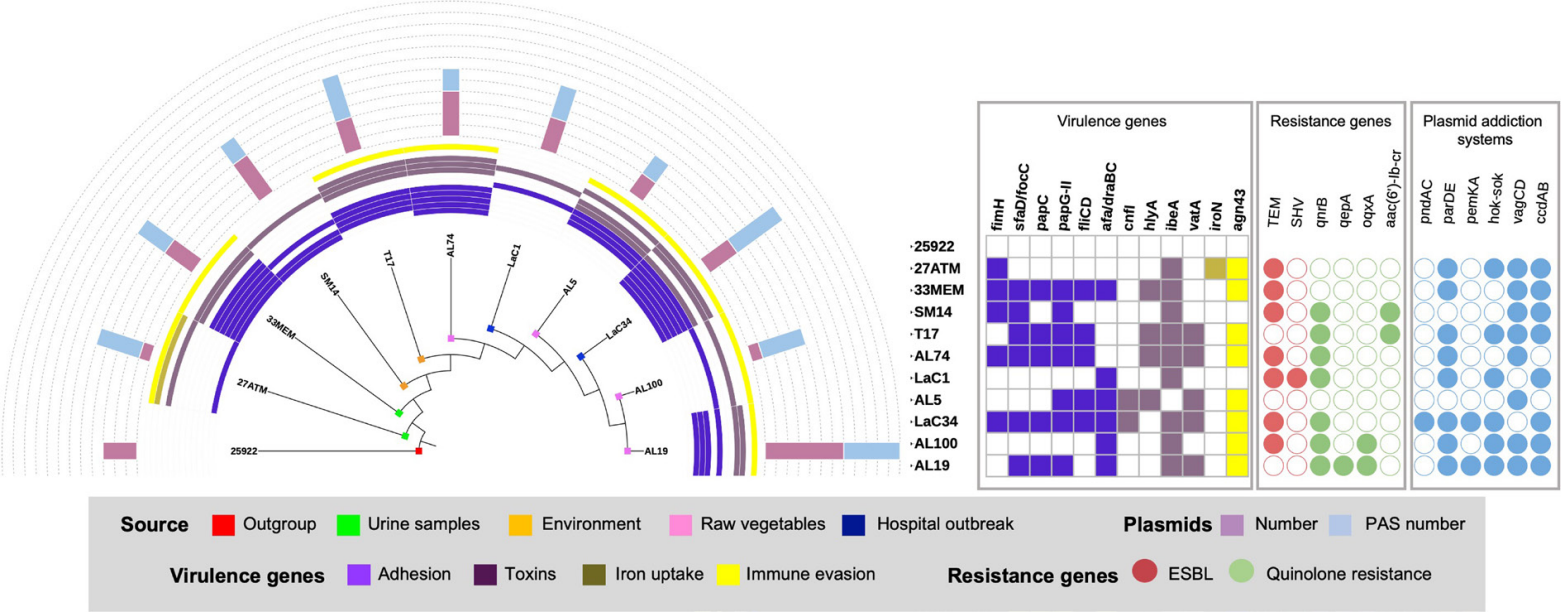
ERIC-PCR-based phylogenetic trees of *L. adecarboxylata* from several sources, and a heatmap showing the presence/absence of 12 virulence genes, six resistance genes, and six PAS. Pink and blue bars indicate the number of plasmids and PAS.

An ERIC-PCR-based phylogeny was performed to establish a phylogenetic relationship among the *L. adecarboxylata* strains investigated here. Two clusters were identified: one comprising strains isolated from raw vegetables and those obtained from a hospital outbreak, and the other, sufficiently distant, comprising strains that include those isolated from polymicrobial urine cultures and environment (Fig. 3). Interestingly, in the group of environmental and urine strains are found the most virulent strains with a similar genetic load of virulence genes with LaC34, which was previously reported as highly pathogenic. Similarly, the most prevalent resistance genes were *bla*_TEM_ and *qnrB*, which were observed in seven strains, one of which also harboured *bla*_SHV_. On the other hand, the gene *qnrB* was observed in seven strains as the more prevalent quinolone resistance-associated gene (Fig. 3).

Virulence and resistance genetic determinants are frequently mobilized by conjugative plasmids which persist in the host through PAS. The more prevalent PAS were ParD-ParE, VagC-VagD, and CcdA-CcdB observed in 80 % (8/10) of the strains; Hok-Sok was presented in 60 % (6/10) of the strains; and finally, PemK-PemA and PndA-PndC were observed only in two and one strain respectively (Fig. 3).

## Discussion

Increasing evidence during the last years reinforce the clinical importance of *L. adecarboxylata* even the low frequency of reports in clinical practice; this may be due to the technical difficulties in non-automated diagnosis by the routine laboratory and the need for tests that are not widely available in a generalized way, such as molecular testing. One of the traits considered to discern between *Escherichia* and *Leclerecia* species is the fosfomycin resistance, interestingly 50 % (5/10) of the strains were sensitive to this drug. However, this behaviour had been previously reported by Zayet *et al*. in a retrospective study conducted in France, in which they reported fosfomycin resistance in 50 % of the nosocomial strains investigated in that study [38].

In the present study, we identified quinolone resistance in two strains (20 %) contrasting to those reported by Akinbami *et al*. in a study performed in Nigeria where they demonstrated resistance to moxifloxacin but sensitivity to ciprofloxacin in a subset of 70 Enterobacterales strains including *L. adecarboxylata* [39]; interestingly, the strains showing quinolone resistance were those associated to a hospital outbreak. In this same Nigerian study, they reported 25 % of ESBL-producing strains. The more prevalent genes associated were *bla*_TEM_ (26 %, *n*=7), *bla*_SHV_ (11.1 %, *n*=2), and *bla*_CTX-M_ (6.67 %, *n*=1) [39], while our samples showed ESBL resistance in 50 % of the strains but 70 % of the strains harboured *bla*_TEM_; interestingly, just one of our strains presented *bla*_SHV_, and none carried *bla*_CTX-M_. In a recent work from a diseased synanthropic pigeon in Brazil using a genomic approach, Sano *et al*. reported a quinolone-resistant and ESBL-producing *L. adecarboxylata* strain but harbouring *bla*_LAP-2_ (a β-lactamase first reported in *Enterobacter cloacae*), and those related to quinolone resistance (*qnrS*, *qnrB* and mutations in *gyrA* and *parC*) [40]. This is consistent with our findings that showed a high presence of *qnrB* in 70 % of our *L. adecarboxylata* strains; an interesting finding was that additionally, the genes *oqxA* (in two samples), *qepA* (in one sample), and *aac(6’)-Ib-cr* (in two samples) also associated to quinolone resistance were identified. In the same way, in Uruguay, a clinical isolate of an *L. adecarboxylata* strain with *aac(6’)-Ib-cr* was reported into a class one integron arrangement. The gene *bla*_SHV_, was located near an *IS*26 insertion sequence [41] so it will be interesting to investigate similar genetic arrangements in our strains in future experiments. The resistance patterns reported here are of importance as previous work has shown limited resistance, such as Sng’s findings of *L. adecarboxylata* strains with pan-sensitive behaviour exhibiting resistance only to cephalothin and trimethoprim/sulfamethoxazole [42]. In the same sense, Gajdács *et al*., observed a similar pattern of pan-sensitivity in a 13 year retrospective analysis in Hungary and a low resistance (just 8 % *n*=3) showed resistance to ampicillin, norfloxacin and nitrofurantoin [43]. Our findings reporting resistance determinants in strains from several sources is important to recognize the emergence of multidrug-resistant strains that can be acquired in the hospital environment and in the community and spread to different niches. Since the previous works reporting the emergence of MDR strains of *L. adecarboxylata,* multidrug resistance in this bacterial species is increasing [13,15, 44,46], therefor, surveillance and reporting of such isolates is of clinical importance and may eventually have epidemiological relevance.

Conversely, we observed a differential distribution of plasmids among the strains ranging from 7 to 125 kbps in co-occurrence with Plasmid Addiction Systems. Interestingly, we observed two strains (27ATM and AL100) with only one plasmid but with four PAS, suggesting redundant mechanisms of plasmid persistence; these findings contrast with those reported by Fang *et al*. in 2016 in which they observed up to three PAS located on IncH2 plasmids from *E. coli* [47]. We observed a higher presence of PAS in strains that produce *bla*_CTX-M_, compared to those that only presented *bla*_TEM_, agreeing with Basma *et al*. in a study conducted in France [20]. Interestingly, we only observed PndAC in strain AL74, and this PAS has been frequently reported in *E. coli* strains isolated from animals such as chickens, pigs and turtles [48,50]. In addition, CcdAB, PemK and Hok-Sok were also observed in AL74 strain; together with VagCD, those three PAS have been reported to be the most representative in clinical samples related to the spread of β-lactamases [51]; however, in two of our clinical samples we did not observe VagCD. ParDE has been found to help modulate the stress response during antimicrobial treatment, confer protection against DNA damage or loss and induce bacterial tolerance [52], which is important in the survival of bacterial biofilms in *Caulobacter crescentus* [53], plasmids related to these PAS might be involved in environmental tolerance and pathogenic potential of *L. adecarboxylata*.

PAS are associated with plasmid stability, allowing plasmids to persist despite bacterial fitness [54]. Reports of PAS associated to *L. adecarboxylata* are scarce, but interestingly, Sun *et al*., reported a *L. adecarboxylata* strain carrying resistance determinants to colisitin (*mcr4*.3) and to imipenem (*bla*_IMP_) harboured in independent plasmids; they also reported the RelE and PemK addiction systems [46]. PAS have been described in conjugative plasmids that often carry virulence and resistance genes that contribute to complicate clinical outcomes, bacterial genomic diversity, and the spread of resistance [49,55, 56]. An interesting perspective would be the characterization of plasmid incompatibility groups in our strains. One of the limitations of this study is the number of strains, nevertheless, it gives insights into virulence potential of this species as well as their contributing role as carriers of resistance genes. Other approaches to elucidate the virulence mechanism could include, among others, the evaluation of *L. adecarboxylata* strains with specific virulence gene mutations/complementation in cell culture or animal models, based on whole genome sequencing of the most promising strains obtained in this work.

*L. adecarboxylata* is an emerging pathogen opportunistic pathogen in polymicrobial infections, immunocompromised patients, and immunocompetent persons. This study suggests that *L. adecarboxylata* strains could play an important role as carriers of virulence and resistance for other bacterial species and therefore their surveillance is of clinical importance.

## supplementary material

10.1099/mic.0.001457 Supplementary Material 1.

## Abbreviations

AMK: amikacin
AMP: ampicillin
BSI: bloodstream infection
CAZ: ceftazidime
CIP: ciprofloxacin
CLSI: Clinical Laboratory Standards Institute
CRO: ceftriaxone
CTX: cefotaxime
CXM: cefuroxime
DCI: cephalotin
ESBL: extended espectrum beta lactamase
ETP: ertapenem
FEP: cefepime
FOS: fosfomycin
FOX: cefoxitin
G: gentamicin
Ie: immune evasion
IU: iron uptake
LB: Luria Bertani
MDR: multidrug resistant
MEM: meropenem
MIC: minimum inhibitory concentration
NIT: nitrofurantoin
NOR: norfloxacin
O.D.: optical density
PAS: plasmid addiction system
PCR: polymerase chain reaction
SAM: ampicillin/sulbactam
TMP-SMX: trimethoprim/sulfamethoxazole
TPN: total parenteral nutrition
WGS: whole genome sequencing
