# Supplementary material for: Resistance phenotype and virulence potential of Leclercia adecarboxylata strains isolated from different sources
Source: Microbiology (Reading). 2024 Apr 25;170(4):001457. doi: 10.1099/mic.0.001457 (PMC11084626; doi:10.1099/mic.0.001457)
Supplement: Supplementary Material 1. [file mic-170-01457-s001.pdf]

Supplementary table 1. Primers used in this study.

Use/target	Primer name	Sequence (5'-3')	Reference
E. coli identification	*ybbW*-F	GTGATTGGCAAAATCTGGCCG	(23)
	ybbW-E	CATACTGGCAATCAGTACGCC	(24)
L. adecarboxylata identification	*glgX*-F	GCACGGTGTATGTGTATTCCAG	(22)
	glgX-R	TCCTCACTGAAGAACGACCAG	
	bamA-F	GCTGATAAACGGCGTAGGGGTA	
	bamA-R	GTTTCTTCCCGACCGAAGGCTC	
ERIC PCR	ERIC-1	ATGTAAGCTCCTGGGGATTCAC	(33)
	ERIC-2	AAGTAAGTGACTGGGGTGAGCG	
bla$_{\text{CTX-M1-8}}$	Fwd	TGTGCAGYACCAGTAARGYKATG	(25)
	Rev	TARRTSACCAGAAYVAGCGGC	
bla$_{\text{CTX-M2}}$	Fwd	CGAGTGGCAGTACCAGTAAGG	
	Rev	CGATATCGTTGGTGGTGC	
bla$_{\text{CTX-M9}}$	Fwd	ATGGTGACAAAGAGAGTGCAA	
	Rev	AATATCATTGGTGGTGCCGTAG	
bla$_{\text{CTX-M151}}$	Fwd	GCGGCCATGATAGGTACG	
	Rev	AAAGTAAGTCACAATAACCAGCG	
bla$_{\text{TEM}}$	Fwd	CAACATTTTCGTGTCGCCC	
	Rev	GCTTAATCAGTGAGGCACC	
bla$_{\text{SHV}}$	Fwd	TATTATCTCCCTGTTAGCCA	
	Rev	CGCTCTGCTTTGTTATTC	
oqxA	Fwd	CTCGGCGCGATGATGCT	(26)
	Rev	CCACTCTTCACGGGAGACGA	
oqxB	Fwd	TTCTCCCCCGGCGGGAAGTAC	
	Rev	CTCGGCCATTTTGGCGCGTA	
qepA	Fwd	GCAGGTCCAGCAGCGGGTAG	(28)
	Rev	CAACTGCTTGAGCCCGTAG	
qnrB	Fwd	GATCGTGAAAGCCAGAAAGG	(29)
	Rev	ACGATGCCTGGTAGTTGTCC	
aac(6')-Ib-cr	Fwd	TTGGCATGTTCTATGAGTGGCTA	(30)
	Rev	CTCGAATGCCTGGCGTGTTT	
oqxA	Fwd	CTCGGCGCGATGATGCT	(26)
	Rev	CCACTCTTCACGGGAGACGA	
oqxB	Fwd	TTCTCCCCCGGCGGGAAGTAC	
	Rev	CTCGGCCATTTTGGCGCGTA	
fimH	Fwd	TTATGGCGGCGTGTTATC	(27)
	Rev	TCCCTACTGCTCCTAACG	
sfaD/focC	Fwd	AGGCAAATGGACAGGTATGG	
	Rev	TCACCCAGAACAAACTTTCC	
papG-II	Fwd	ATTCACCATAGAGGCGACTG	
	Rev	ATCATTATGCGGCTCAGAC	
papC	Fwd	TTCTCTCTCCCTCAATACGG	
	Rev	TTATAACCTCAACGGGACGG	
fliCD	Fwd	CCGAATCAGAGTTAGTTCCG	
	Rev	CCCAGCGATGAAATACTTGC	
sat	Fwd	GTTGGCAAACAGGTCAAAC	
	Rev	CTCGGAGTATTGGCTTCAG	
hlyA	Fwd	GATACGCTGATAGGTGAG	
	Rev	CCAGGTGTGACTCAATAC	
kpsM	Fwd	CCAGAGTAGATATGACCAG	

	Rev	CTACGAGAAATACGAACAC
agn43	Fwd	CACACAGCCACTAATACC
	Rev	CACCTGAATACCCTTACC
vat	Fwd	ATACAGTCTCGTCTCTGG
	Rev	GTGACAGTCCCTTTATCC
cnf-1	Fwd	CAGACTCATCTTCACTCG
	Rev	AGACAGAGACCTTACGAC
traT	Fwd	TGGTATAGTTCACATCTTCC
	Rev	TAAAGCCTACTACTGGATTC
fyuA	Fwd	CGCCAGTAAACAATCTTCCC
	Rev	CCCAAACACCATATCAACGG
iucD	Fwd	CGTGAGACCCAGTTTATTTCC
	Rev	GGGCTGCTGAAGATATGAATAACC
iroN	Fwd	CAGAATGATGCGGTAACTCC
	Rev	CGTGAGACCCAGTTTATTTCC
iutA	Fwd	GTTCACGCTCTTTGTCAGG
	Rev	GGGCTTAATCTCGGGAAAGG
feoB	Fwd	GTCTAACCTTGAGCGTAACC
	Rev	GGCGAGGAAGATAGTCAGC
iha	Fwd	TGTGCTCTGGTTTGATATGG
	Rev	CATTCTGGGTGCCTTATATCC

Supplementary Table 2. Minimum Inhibitory Concentration (mg/mL) of the *L. adecarboxylata* strains.

Strain	AMP	SAM	DCI	CXM	CTX	CAZ	CRO	FEP	ETP	MEM	AMK	G	CIP	NOR	FOS	NIT	TMP-SMX
LaC1	≥32 (R)	≥32 (R)	≥64 (R)	≥64 (R)	≥64 (R)	≥64 (R)	≥64 (R)	16 (R)	≥8 (R)	≥16 (R)	4 (S)	≤1 (S)	2 (R)	2 (S)	≥256 (R)	≤16 (S)	≥320 (R)
LaC34	≥32 (R)	≥32 (R)	≥64 (R)	≥64 (R)	≥64 (R)	≥64 (R)	≥64 (R)	16 (R)	≥8 (R)	≥16 (R)	≤2 (S)	≤1 (S)	2 (R)	2 (S)	≥256 (R)	≤16 (S)	≥320 (R)
AL5	≥32 (R)	4 (S)	≤2 (S)	2 (S)	≤1 (S)	≤1 (S)	≤1 (S)	≤1 (S)	≤0.5 (S)	≤0.25 (S)	≤2 (S)	≤1 (S)	≤0.25 (S)	≤0.5 (S)	≤16 (S)	64 (I)	≤20 (S)
AL19	≤2 (S)	≤2 (S)	≤2 (S)	≤1 (S)	≤1 (S)	≤1 (S)	≤1 (S)	≤1 (S)	≤0.5 (S)	≤0.25 (S)	≤2 (S)	≤1 (S)	≤0.25 (S)	≤0.5 (S)	≤16 (S)	≤16 (S)	≤16 (S)
AL74	≤2 (S)	≤2 (S)	≤2 (S)	≤1 (S)	≤1 (S)	≤1 (S)	≤1 (S)	≤1 (S)	≤0.5 (S)	≤0.25 (S)	≤2 (S)	≤1 (S)	≤0.25 (S)	≤0.5 (S)	≥256 (R)	≤16 (S)	≤20 (S)
AL100	8 (S)	4 (S)	≥64 (R)	4 (S)	4 (S)	≤1 (S)	≤1 (S)	≤1 (S)	≤0.5 (S)	≤0.25 (S)	≤2 (S)	≤1 (S)	≤0.25 (S)	≤0.5 (S)	≤16 (S)	≤16 (S)	≤16 (S)
27ATM	4 (S)	≤2 (S)	8 (S)	4 (S)	≤1 (S)	2 (S)	≤1 (S)	4 (S)*	4 (R)	≤0.25 (S)	4 (S)	≤1 (S)	≤0.25 (S)	≤0.5 (S)	≤16 (S)	≤16 (S)	≤20 (S)
33MEM	≤2 (S)	≤2 (S)	32 (R)	≥64 (R)	≤1 (S)	≥64 (R)	≥64 (R)	8 (S)*	4 (R)	≥8 (R)	4 (S)	≤1 (S)	0.5 (I)	1 (S)	≥256 (R)	≤16 (S)	≤20 (S)
SM14	16 (I)	≤2 (S)		≥64 (R)	≤1 (S)	≤1 (S)	≤1 (S)	≤1 (S)	≤0.5 (S)	4 (R)	≤2 (S)	≤1 (S)	≤0.25 (S)	≤0.5 (S)	≤16 (S)	32 (S)	≤20 (S)
T17	≤2 (S)	≤2 (S)	4 (S)	≤1 (S)	≤1 (S)	≤1 (S)	≤1 (S)	≤1 (S)	≤0.5 (S)	≤0.25 (S)	≤2 (S)	≤1 (S)	≤0.25 (S)	≤0.5 (S)	≥256 (R)	≤16 (S)	≤20 (S)

AMP, ampicillin; SAM, ampicillin/sulbactam; DCI, cephalothin; CXM, cefuroxime; CTX, cefotaxime; CAZ, ceftazidime; CRO, ceftriaxone; FEP, cefepime; ETP, ertapenem; MEM, meropenem; AMK, amikacin; G, gentamicin; CIP, ciprofloxacin; NOR, norfloxacin; FOS, fosfomycin; NIT, nitrofurantoin; TMP-SMX, trimethoprim/sulfamethoxazole. *DDS, dose-dependent sensitivity. S, sensitive; I, intermediate sensitivity; R, resistant.

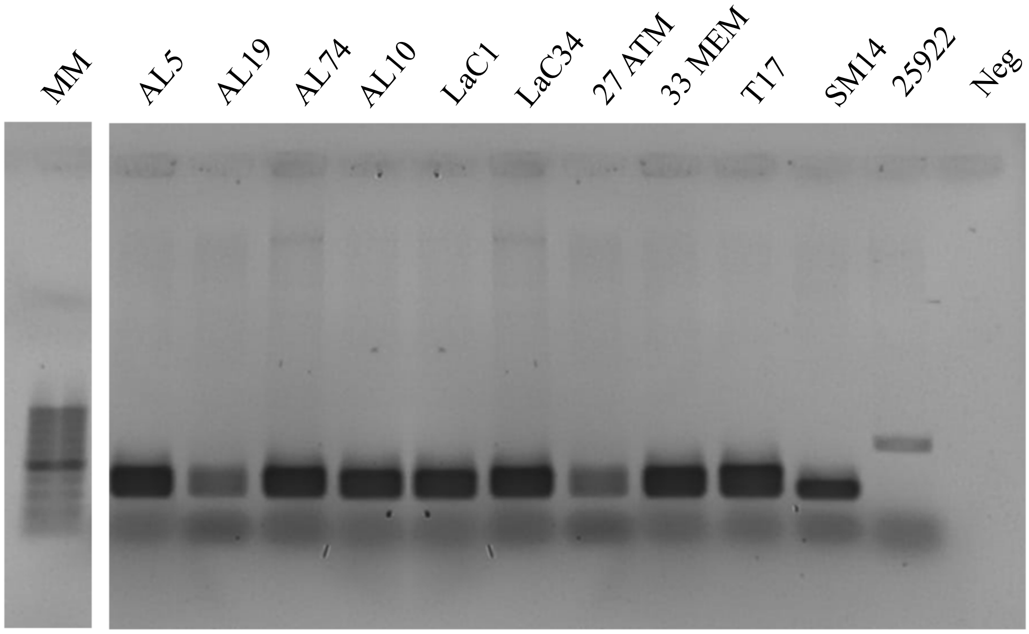

Supplemental Figure 1. 1% agarose gel electrophoresis of the PCR products for molecular identification amplifying specific genes: *ybbW* (667bp), *glgX* (327bp) and *bamA* (419bp). MM:100bp molecular weight marker; Neg: negative control; 25922: *E. coli* ATTC25922 used as control.

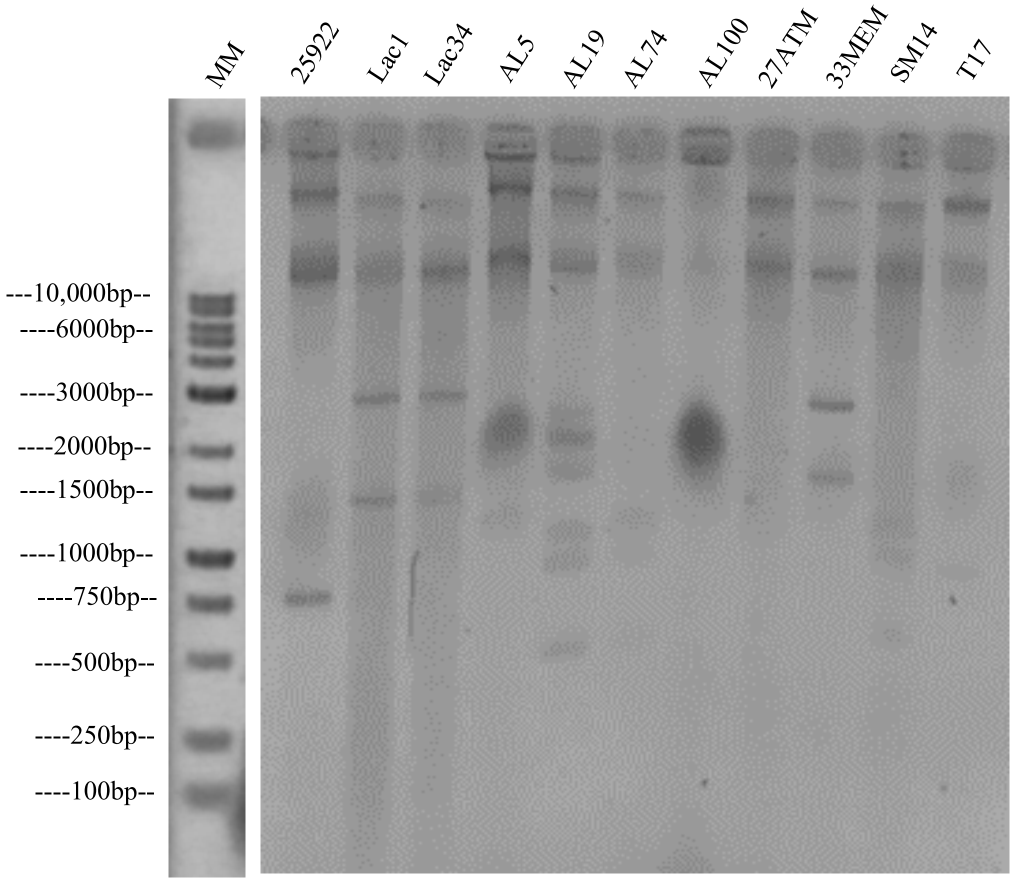

Supplemental Figure 2. 1% gel electrophoresis of the plasmid extraction. *E. coli* ATCC25922 was used as control.
